# Design and validation of AQUA CHILD—Pre‐aquatic questionnaire assessing child development

**DOI:** 10.1002/brb3.70033

**Published:** 2024-09-29

**Authors:** Merav Hadar Frumer, Huib Ten Napel, Maria José Yuste‐Sánchez, Isabel Rodríguez‐Costa

**Affiliations:** ^1^ Israel Sport Centre for the Disabled (ISCD) Ilan Spivak Ramat Gan Israel; ^2^ University of Alcalá Alcalá de Henares Spain; ^3^ Physiotherapy in Women’s Health Research Group – FPSM, Department of Nursing and Physiotherapy University of Alcalá, Alcalá de Henares Madrid Spain; ^4^ Ramón y Cajal Institute of Health Research ‐IRYCIS University Hospital of Ramón y Cajal Madrid Spain; ^5^ Humanization in the intervention of physiotherapy for the integral attention to the people (HIPATIA), Physical Therapy Degree, Department of Nursing and Physical Therapy Universidad de Alcalá, Alcalá de Henares Madrid Spain

**Keywords:** aquatics, assessment, children, developmental delay, disability and health: children and youth version (ICF‐CY), The International Classification of Functioning

## Abstract

**Purpose:**

We developed a proxy questionnaire for parents of children with Developmental Delay (DD) to provide comprehensive information for instructors about the child's functioning before participating in aquatic activities. This dedicated information will enable a high‐quality treatment plan to promote the child's functioning in everyday life.

**Methods:**

Based on the International Classification of Functioning, Disability, and Health (ICF) Coreset development and linking rules method, a set of questions was constructed in a preliminary process. A draft version was sent to instructors and parents in Israel. Seventy‐five questionnaires from instructors and 25 from parents returned to the statistical analysis procedure. Reliability and face validity were analyzed by experts.

**Results and conclusions:**

The questionnaire showed high validity and reliability for its purposes and allowed self‐completion by the parents.

## Graphical abstract

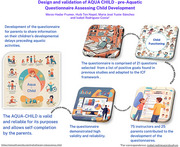

## 1 INTRODUCTION

For decades, the aquatic environment (AE) has been designated for purposes of everyday life activities including swimming, relaxation, and physical fitness. Many aquatic activities (AA), focused on promoting human capabilities and rehabilitation, have been developed and studied under general names like “Aquatic therapy” “Hydrotherapy” and many more. Aquatic‐based activities are conducted by an array of professionals (Hadar‐Frumer et al., 2023; Moffatt, 2017). These “instructors” (a generic name for all professionals working in the AE within the Halliwick approach) (Barrett & Maes, 2021; Gresswell et al., 2010) utilize the aquatic properties (i.e., hydrostatic pressure, buoyancy, turbulence, viscosity, temperature, etc.), along with special aquatic techniques and approaches developed specifically for all kinds of AA (Becker, 2020).

Aquatic Activity targets promoting children's abilities in everyday life. It is always focused on setting a quality program, based on the child's abilities and the parents' expectations (Chandolias et al., 2022; Grosse, 2011; Hamed et al., 2023).

Working with children in the AE has proved to be effective in many activities and swimming skills. In some cases, the AE is the only environment where individuals with severe impairments can move and practice active movement that cannot be practiced on land (Bairaktaridou et al., 2021; Brokaw, 2022; Güeita‐Rodríguez et al., 2021; Güeita‐Rodríguez et al., 2018; Güeita‐Rodríguez et al., 2017; Muñoz‐Blanco et al., 2020; Tapia et al., 2023; Trisnowiyanto & Syatibi, 2020; Vaščáková et al., 2015; Vodakova et al., 2022).

In recent years, many articles attributed the investigated treatment plan within the AE, to the domains of the International Classification of Functioning, Disability and Health (ICF), a biopsychosocial model of the World Health Organization (WHO) (WHO, 2002; WHO, 2007). These researchers are working toward the aim of creating a uniform language for all purposes—treatment, research, publications, and professional jargon, within the AE and on land (Cross et al., 2013; Cuesta‐Vargas et al., 2020; Gorter & Currie, 2011; Güeita‐Rodríguez et al., 2017; Güeita‐Rodríguez et al., 2019; Khalaji et al., 2017; Vaščáková et al., 2015).

Quality of life is a comprehensive and important concept in every person's life (Hernández‐Segura et al., 2022; McDougall et al., 2011; Rosenberg et al., 2011; Simeonsson, 2009; Suárez et al., 2018; The World Health Organization Quality of Life Assessment (WHOQOL) 1998). The WHO defined it as “subjective well‐being.” It involves the individual's perception of his/her position in life within the context of his/her environment. It also involves one's own life goals and his/her feelings about the influence of the Health Condition on their life (Hadar‐Frumer et al., 2023; McDougall et al., 2011; Rosenberg et al., 2011; Simeonsson, 2009; Suárez et al., 2018; The World Health Organization Quality of Life Assessment (WHOQOL), 1998, WHO, 2002; WHO, 2007).

There are many questionnaires and assessment tools for the field of quality of life. These questionnaires usually refer to two main areas: specific—to specific diagnostic groups or general—to different types of diagnoses at different levels of severity and to the entire population (Hernández‐Segura et al., 2022).

Most of the questionnaires refer to three main areas in defining the quality of life:

Health status—functional ability and general health;

Quality of life (QoL)—regarding the person's needs, expectations, and feeling of satisfaction with his/her life and the social situation in accordance with his social and cultural values;

HRQOL—a complex definition that mainly refers to the various areas in a person's life that are affected by their health status with various indicators such as the assessment of a person's wellbeing considering their mental and social condition, the assessment of their condition only in matters related to their health, the assessment of the factors affected by their health condition and how they affect quality his life and the like (Hadar‐Frumer et al., 2023; Hernández‐Segura et al., 2022; Karimi & Brazier, 2016).

QoL still does not have a structured and clear definition within the ICF model; however, it is directly linked to similar concepts such as subjective well‐being and overall life satisfaction (WHO, 2002; WHO, 2007; Hadar‐Frumer et al., 2023).

When the AA is focusing on promoting the child's QoL it requires the instructor's ability to identify the children's needs in the two environments in which they operate—their everyday life (land) and their AE activities (water) (Israel & Pardo, 2014).

There are many land‐based measurement tools for assessing a child's ability in various areas of daily life (Cuesta‐Vargas et al., 2020; Israel & Pardo, 2014). When it comes to the aquatic activity assessment, the tests and questionnaires that are available at present, only assess the aquatic capabilities of the child (Güeita‐Rodríguez et al., 2019; Kokaridas & Lambeck, 2015), so professionals are using methodologies involving assessment out of the water with validated land‐based scales or instruments (Israel & Pardo, 2014). The difficulty in using these kinds of tools to assess the children's general abilities and QoL is because AE is an environment with unique properties such as hydrostatic pressure, buoyancy, resistance to movement, heat, and more (Hadar‐Frumer et al., 2023; Israel & Pardo, 2014; Becker, 2009). This environment allows children a different motor experience than on land. Many times, this experience is easier due to these properties, and the ability to perform an activity is made possible by the support of the water, reducing the forces of gravity on the body, reducing pain and muscle tone, and the possibility of creating work on strength without excessive load on the skeleton (Hadar‐Frumer et al., 2023; Israel & Pardo, 2014; Becker, 2009). For these reasons, there is difficulty in comparing the effect of the AA on the Qol of the child in the AE compared to the land environment and it is necessary to find tools that will accurate the assessment and make it easier for the professionals to choose appropriate work goals for both environments (Hadar‐Frumer et al., 2023; Israel & Pardo, 2014).

To date, to our knowledge, there is no evaluation tool, aiming at AA for children, which can provide unified information for both environments and will allow the evaluator to determine goals for both—the aquatic exercises and the child's daily life activities.

For that reason, we developed a new questionnaire, based on the ICF‐CY (ICF for Children and Youth) framework, aiming to provide all professionals working within the AE with children with developmental delays (DD) aged 6–12, a clear and concise measure of the child's abilities. It will allow these professionals to establish functioning goals based on one unified assessment, related to the QOL of these children and their functioning in everyday life. These goals will define literally and practically the activities on which the AA plan should focus.

### 1.1 Why an ICF‐CY‐based questionnaire?

**FIGURE 1:**
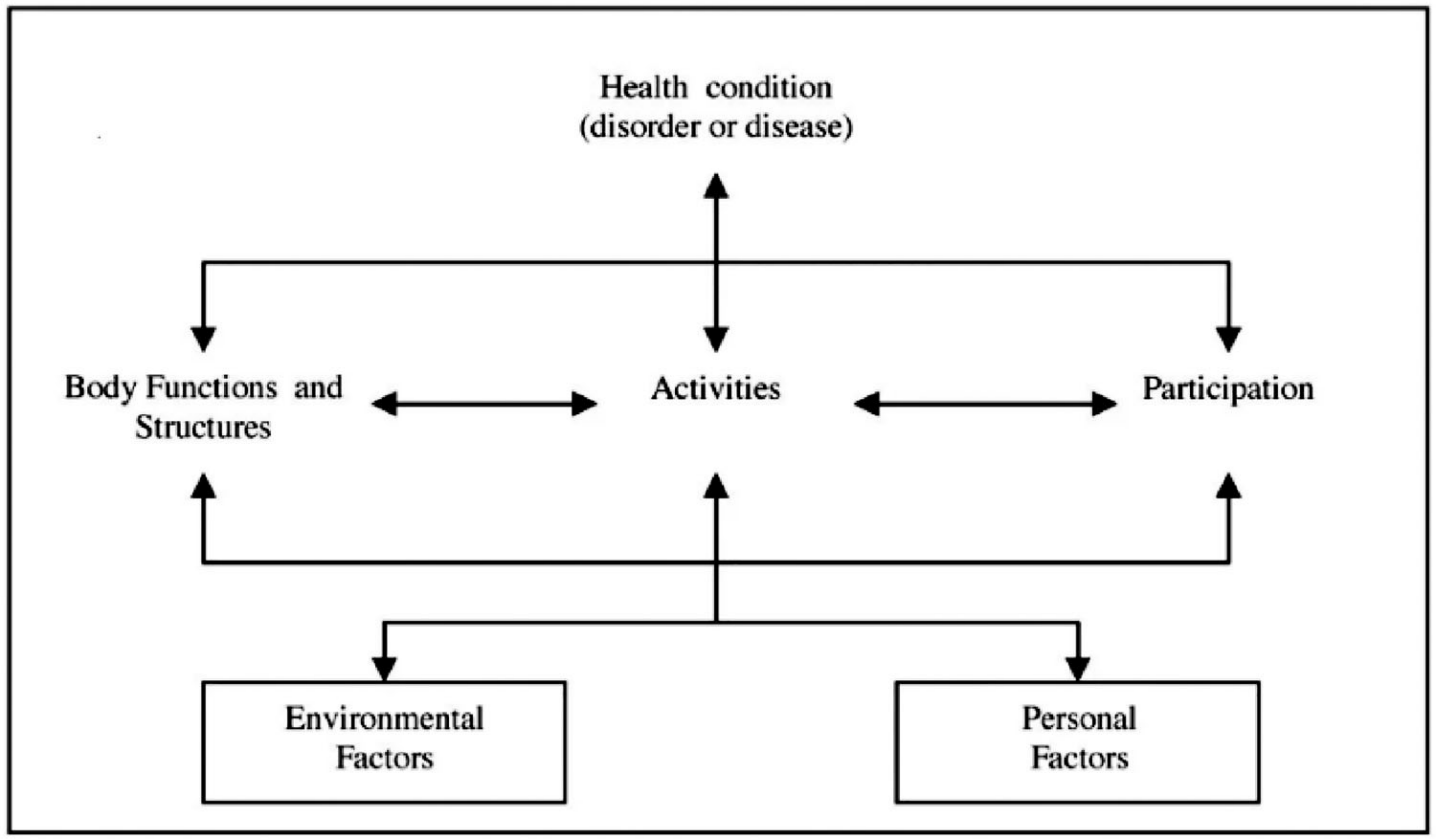
The ICF framework: Interaction between ICF components (WHO, 2002; WHO, 2007).

The ICF was developed by the WHO to provide a standardized language and comprehensive model for understanding health and functioning through six main components (Figure 1).

The ICF‐CY stands for the ICF for Children and Youth. It includes additional categories lacking in the ICF which are specifically relevant to children and youth. It places particular importance on collaboration with families and involving them as active participants in all activities, as they influence all aspects of the child's development and well‐being (WHO, 2007; Simeonsson, 2009). It is one of the leading models today which allows the professionals to refer to all aspects of the child's functioning and lifestyle, which are the starting point for the rehabilitation process. Specific constructs such as “gaps” and “delays” in the child's development are also included, which are important for the rehabilitation process. It “provides a context for identifying the relationship among measures used in multidisciplinary assessment” [38 p. 347].

The ICF framework was found to be a valid and reliable tool for evaluating children leading to a better understanding of their needs and to determine a better treatment plan (Güeita‐Rodríguez et al., 2019; Jelsma & Scott, 2011; Illum & Gradel, 2017; Franki et al., 2014). It allows for systematic data collection and a full description of the child in the levels of activities and participation (A&P) components and supports better communication between the various professionals and parents (Watter et al., 2008; Illum & Gradel, 2017; Grill & Stucki, 2009; Rauch et al., 2008).

The instructors, working in the AE, can use the ICF framework to set goals regarding all areas of rehabilitation as well as reaching independent swimming and using that skill to be active, participate in community life, and improve QoL (Moffatt, 2017; Barrett & Maes, 2021; Bairaktaridou et al., 2021; Güeita‐Rodríguez et al., 2021; Güeita‐Rodríguez et al., 2018; Vaščáková et al., 2015; Güeita‐Rodríguez et al., 2019; Hadar‐Frumer et al., 2023).

A questionnaire using the ICF‐CY domains will unify the two different environments (land and AE). It can serve as a single assessment and goal‐setting tool before the children enter AA (Güeita‐Rodríguez et al., 2017; Vaščáková et al., 2015; Güeita‐Rodríguez et al., 2019; Khalaji et al., 2017; Cross et al., 2013; Rauch et al., 2008).

### 1.2 Developmental delay

The questionnaire is based on the principles of the ICF‐CY, which is an important construct in the functioning of children; therefore, we strive for the questionnaire to be adapted to the widest number of children and allow a clear reference to the quality of the child's life regardless of his/her health status or diagnosis.

Developmental delay (DD) is a descriptive term and not a diagnosis. The appearance of a delay or limitation in a child's functioning is not permanent and can depend on changes over time, the growth and general development of each child. DD is a broad term that describes the lag in the child's development in one or more of these various domains: gross and fine motor skills, speech and language, social and personal skills, activities of daily living, and cognition (World Health Organization, 2007; Brian et al., 2019; Choo et al., 2019; Tonelli et al., 2016). It also depends on the age of the child and his emotional state. This is the reason why in ICF‐CY there is an inclusive reference which is reflected in the word “delay” which enables the assessment and documentation of the child's functions according to the various changes over time (World Health Organization, 2007).

A child diagnosed with DD is a child who does not achieve the developmental milestones that children in his/her age range usually achieve (Choo et al., 2019).

The child's level of development can be classified as Mild, Moderate, or severe in accordance with the child's chronological age (Choo et al., 2019).

It is important to note: “Not all children with developmental delay will have a developmental disability, which refers to severe, lifelong impairment in areas of development that affects learning, self‐sufficiency, and adaptive skills. Developmental delays can be transient, such as during a phase of prolonged illness, or persistent” [46, p. 119].

### 1.3 Why the age group of 6–12 years old?

This age group of 6–12 years old (“middle childhood”; Maccoby, 1984) was selected since these children are at elementary school and have similar learning and social requirements as well as similar developmental characteristics. This resemblance enables the researchers to set similar functioning goals.

### 1.4 Why A&P categories?

AA for children is associated with active exercises and participation in various games and activities. Swimming is an important goal for activity, and at the same time, various studies identify a prominent effect of AA on categories of the A&P components of the ICF‐CY in all aspects of life (Muñoz‐Blanco et al., 2020; Trisnowiyanto & Syatibi, 2020; Vaščáková et al., 2015; Khalaji et al., 2017; Dow, 2016). Adolfsson et al. (2018) focus on participation as an important factor for defining the intervention's goal and for evaluating its success. Since participation and engagement require connection and initiative processes, they place high demands on the child, thus promoting his/her development processes.

## 2 THE AIMS OF THE STUDY

The overall objective is to assess whether the new questionnaire can serve as a reliable and valid tool for AA in Israel (a pilot trial), for the initial assessment of the abilities of children with developmental delays, aged 6–12 participating in various aquatic activities. The questionnaire aims to provide comprehensive information about the child and will allow planning of the activity and setting of appropriate goals.

Specified objectives:

‐ *Validation of the questionnaire* (“Face validity” of experts—professionals and parents/ caregivers).

Purposes:

‐ To examine whether the questionnaire is suitable for its purpose—comprehensive and refers to all the important aspects of the child's everyday life.
‐ To find out whether the questionnaire is easy to use.
‐ To find out whether instructors can use the questionnaire to determine aquatic goals and exercises for the aquatic program.
‐ *Internal and interrater reliability of the questionnaire*.

Whether the questionnaire is reliable enough to provide information about the child in terms of:

‐ Do the parents/caregivers provide the same information in two different situations when they answer the questionnaire?
‐ Can the parents/caregivers fill out the questionnaire independently without prior guidance from an instructor?

## 3 METHODS

The preparation and construction of the questionnaire and the research are performed as described in the following processes (Table 1):

**TABLE 1:** Methods in a chronological manner.

Step	Aims	Performed by
**1. Preparatory stage**		
Analysis of studies that examined the effect of AA on children aged 6–12 with DD (Hadar‐Frumer et al., 2023)	A general understanding of the importance of AA for these children An extraction of the goals in which AA has a proven effect	The authors of this article
The procedure of linking the goals that were found to be positive to the ICF‐CY definitions (Hadar‐Frumer et al., 2023)	Examining whether it is possible to use the ICF‐CY terms as a tool for all the goals extracted from the studies Establishing an initial database of goals from which the questions for the questionnaire will be selected	HTN and MHF
**2. Selecting the categories for the questionnaire—a Delphi consensus procedure** (Duffield, 1993)	Choosing the significant questions for the questionnaire	11 experts in the field of AA from various professions, then, a review and approval by the authors of the article
**3. Constructing of the questionnaire** (incl. obtaining Helsinki approval in Israel)	Arranging the questionnaire and determining the research questions for the instructors and parents	The authors of this article
**4. Translation of the questionnaire into Hebrew. Adapting it to everyday language**	To adapt the questionnaire to the Israeli population To make it accessible for people who are not experts in the ICF‐CY language	2 specialists in translation from English to Hebrew and back Adapting—by MHF
**5. Distribution of the questionnaire to instructors and parents**	Before distribution—a short training process for instructors in Israel. Distributing the questionnaire to these instructors and returning the completed questionnaires to MHF in the same way.	MHF
**6. Statistical analysis of the instructors' and parents' answers**	For the questionnaire's reliability and face validity	An expert in statistical analysis
**7. Adjusting the questionnaire—adding and removing questions according to the responses of parents and instructors**	Adapting the questionnaire precisely to the needs of parents and instructors	The authors of this article

### 3.1 Elaboration and explanation of the methods

1. Preparatory stage—A systematic review and extraction of the questionnaire's preliminary list of A&P components. The method of the preparatory stage was based on the ICF Coreset development and linking rules method as used by Postma et al. (2018).
  ‐ A preliminary study procedure (Hadar‐Frumer et al., 2023) was carried out in which 71 articles, published between 1.1.2010 and 31.1.2020, were reviewed for the positive effects of AA on children with DD of primary school age (Hadar‐Frumer et al., 2023).
  ‐ A procedure of linking the positive goals found in the selected articles, to the ICF‐CY definitions. The 443 different positive goals that were found underwent an ICF‐linking process (according to the ICF linking rules; Cieza et al., 2019) by the leading researcher (MHF) and an ICF expert (HTN). After 8 sessions of an iterative consensus process, 270 different ICF‐CY categories were obtained, on which AA had a positive effect, 138 of which belonged to the A&P components (Hadar‐Frumer et al., 2023). These categories formed the preliminary list for choosing the questions that made up the questionnaire.

2. Selecting the categories for the questionnaire—a Delphi consensus procedure (Duffield, 1993)
  ‐ For the process of selecting the categories, applications were sent to 20 Halliwick lecturers worldwide. In recent years, the International Halliwick Association has expressed great interest in integrating the ICF into its working principles and developing tools for aquatic assessment that combine the principles of the Halliwick approach and the ICF (Barrett & Maes, 2021; Kokaridas & Lambeck, 2015; Rohn et al., 2021; Lieto et al., 2022). The Halliwick approach is unique in being an approach that promotes swimming instruction that includes play and fun activities, and many of the instructors within this approach use its 10‐point program for the treatment and rehabilitation of children (Gresswell et al., 2010; Barrett & Maes, 2021).
  ‐ Ten lecturers from six different countries joined the main researcher (MHF) and expressed their agreement to participate in the process. The experts came from Israel, Poland, Croatia, Denmark, Spain (Catalonia), and Brazil. All work with children. Of these, six are physiotherapists, one occupational therapist, two physical education teachers, an expert in Chinese medicine, and a swimming coach. All are aquatic therapists and some are certified swimming instructors.
  ‐ In a Delphi consensus process (Duffield, 1993), these experts selected the list of ICF‐CY's A&P categories that they believe are the most relevant for working in the AE with children aged 6–12 with DD.

### 3.2 Predetermined working principles for the Delphi process

‐ Excluding a category requires the consent of all experts.
‐ The questionnaire will include 20–25 questions about children's abilities in the A&P ICF‐CY component (besides the general information questions). This decision was based on the personal experience of the experts and previous researchers' recommendations (Postma et al., 2018; Sharma, 2022; Minto et al., 2017; Setia, 2017).

### 3.3 The Delphi (Duffield, 1993) procedure consisted of five stages

‐ *First stage*—the experts were asked to select 20 categories from the 138 ICF‐CY A&P domains, obtained in the preliminary review of the articles and the link to the ICF‐CY. The experts could also add 5 categories that were not studied before and which, in their opinion, are important to the questionnaire. At the end of this stage, 102 different categories were selected.
‐ *Second stage*—the main researcher (MHF) referred to each of the selected categories and expressed her opinion on whether it should remain. The experts were asked to respond if they agreed and to justify their opinion. At the end of this phase, the experts reached a common agreement to reduce 58 categories. Forty‐four categories remained for which different opinions were expressed.
‐ *Third stage*—the experts received the remaining 44 categories with the opinions written by everyone in the previous stage. The experts were asked to refer to their partners' reasons and decide which categories would be included in the questionnaire (up to 20 categories) and which would be excluded. After this step, 34 categories remained.
‐ *Fourth stage*—the same procedure of persuasion between the experts as in the third step, ended up with 20 categories that the experts agreed upon.
‐ *The fifth stage*—was performed by the authors of the article. They were presented with the categories chosen by the experts and had to decide on the final list by discussing whether to expand a category, combine it with another, or replace it with a similar one. One category was added. The 21 categories finally selected for the questionnaire were agreed upon by all the authors.

3. Constructing of the questionnaire

‐ After agreeing on the relevant categories, the questionnaire was designed existing of three parts:
  1. General information about the child—family, social life, school, basic abilities in the AE, and personal characteristics.
  2. Information about the child's activities in everyday life—21 questions made up of categories selected from the A&P components of the ICF‐CY.
  3. Summary of the information and determine intervention goals for AA (aimed at the child's daily life).

For the research, three additional parts were added:

⇒ Questions for the parents regarding the relevance of the questionnaire to the child's evaluation procedure, including the nature of the questions.
⇒ Questions for instructors regarding the relevance of the questionnaire to the child's evaluation process, including two additional issues: (a) the nature of the questions and (b) the duration of its completion.
⇒ Information about the instructor and his/her main areas of activity in AE.
  4. Translation into Hebrew and adaptation to everyday language
  ‐ The questionnaire was translated into Hebrew according to the WHO's guidelines (World Health Organization, 2018) and was adapted to everyday language.
  5. Distributing the questionnaire and receiving the answers
  ‐ A training process—before distributing the questionnaires, an explanation and training process were carried out for the audience of instructors working in AE in Israel. They learned the basic principles of the ICF‐CY and went over the questionnaire and its goals. The process was carried out through face‐to‐face lectures at aquatic therapy centers and Zoom meetings.
  ‐ Then, the questionnaires, and a link to the questionnaires on Google Drive, were sent by email to all the instructors who participated in the training process. They were asked to complete the questionnaire and pass it on to the parents or caregivers who accompanied the children to the pool activity.
  ‐ The answers from the instructors or parents were received by the main researcher MHF, via Google Drive, personal delivery, or email.
  ‐ For convenience, we name the interviewed group “instructors” and the self‐fill‐out group “parents.”

### 3.4 The questionnaire fill‐out procedure

‐ Interview of the parents/caregivers by the instructor.
‐ Parents/caregivers self‐filling—was carried out several days before the interview with the instructor. Parents of children with DD and caregivers from Israel, who accompany their child to AA and agreed to fill out the questionnaire themselves, received an initial explanation from the instructors about the purpose of the questionnaire. Also, within the introduction of the questionnaire, there was a detailed explanation of the structure of the questionnaire its objectives, and how to fill it out. The parents were also invited to call MHF for any other explanation (no such request was received). When filling out the questionnaire, no instructor was present with the parents and no direct guidance was given.

### 3.5 Selection of participants and consent to participate in the research

‐ No preselection or definition of this parent's group was made; everyone who volunteered to answer the questionnaire in these two situations entered the statistical analysis.
‐ All respondents were informed, in the explanation part of the questionnaire, that it was used for research purposes and that answering the questions constitutes consent to participate in the research.

6. Statistical analysis
  ‐ *The questionnaire's face validity* was evaluated via three questions about the questionnaire's ability to fit its purpose (parents and instructors), how easy it is to follow (parents) or explain (instructors) the questionnaire, and the ability to derive goals from the questionnaire (instructors only). In all three questions, the options for answers ranged from 1 (to a very large extent) to 5 (not at all).
  ‐ *The questionnaire's reliability*—is the questionnaire reliable enough to provide similar answers from the parents in two situations: (1) in a self‐filling; (2) during an interview with the instructor. To do so, the 25 parents’ questionnaires were compared to those filled out by the same people during the interview with the instructors (“instructors”).
  ‐ The first step of reliability analysis consisted of an evaluation of the scale's Part 2 (21 items detailed in Table 4) internal reliability using Cronbach's alpha. The questionnaire was administered twice—once by the parents and once by the instructors. Both two informers had the same questions. Separate reliability analysis calculation was made for instructors and parents. Although the sample size is small (< 100), Cronbach's alpha was used as the mean between‐scale items correlation is strong (>.5) (Akoglu, 2018). Considering the between‐items associations, it has been suggested that a sample size of 30 can measure reliability using Cronbach's alpha (Conroy, 2015).
  ‐ Next, the intraclass correlation coefficient (ICC) of parents' and instructors' scores was calculated for the questionnaire's parts 2 and 3.
  ‐ Finally, differences between parents and instructors in total scores were evaluated using paired *t*‐tests.
  ‐ The research was approved by the Ethics (Helsinki) Committee in Chaim Sheba Medical Center, Israel, Number 6504‐19‐SMC.

## 4 RESULTS

We developed a questionnaire for instructors in Israel (a pilot trial), for initial assessment of the abilities of children with DD, aged 6–12 participating in various AA. It is intended to provide comprehensive information about the child and to allow the planning of a quality AA program (Annex 1).

The questionnaire was found to have high “face” validity and is suitable for its purposes—receiving comprehensive information about the child, easy to explain and follow, and allowing setting AA goals. It is understandable and allows the parents to provide consistent answers in both situations—by self‐filling out and by answering the instructors’ questions, thus, the instructors can pass the questionnaire to the parents for self‐filling out, preceding the evaluation meeting. As for goal setting, the issue is complex and requires professionalism in the field; therefore, this role must be performed by the instructors and in alignment with the parents.

### 4.1 Detailed results

#### 4.1.1 Participants in the study

A total of 75 children (age range: 6–12 years old; mean age—8.34 ± 1.71; girls, *n* = 31) participated in the study. The children's major abilities in walking and maintaining body positions are detailed in Table 2. Seventy‐five primary caregivers (mothers: *n* = 62, fathers: *n* = 6, others: *n* = 7) and aquatic instructors participated in this study (information about the characteristics of the instructors is given in Table 3). For 25 children from this group, an additional self‐filling questionnaire was filled out by the parents/caregivers before the interview with the instructor.

**TABLE 2:** The children's walking and main postural control abilities (*N* = 75).

Abilities/activities	Walking forward—5 steps	Walking backward—5 steps	Remaining standing	Remaining seated	Maintaining a supine position
Independent	61	52	63	65	71
With support	8	13	11	9	4
Not able/not relevant	6	10	1	1	

**TABLE 3:** Instructors' professional characteristics (*N* = 75).

	Variable	*N* (%)
**Profession**	Aquatic therapist	41 (54.66)
	Aquatic therapist + additional physical education certificate	12 (16.0)
	Aquatic therapist + swimming instructor/ coach	12 (16.0)
	Aquatic therapist + health professions certificate	4 (5.33)
	Aquatic therapist + other certificates	4 (5.33)
	Swimming couch	2 (2.66)
**Years working in the aquatic environment**	First year	8 (10.7)
	1–5 years	9 (12)
	6–10 years	8 (10.7)
	11–15 years	11 (14.7)
	16–20 years	16 (21.3)
	21–25 years	9 (12.0)
	26–30 years	13 (17.3)
	31–35 years	1 (1.3)
**Type of aquatic activity**	Individual Group	54 (72.0) 1 (1.3)
	Individual and group	20 (26.7)
**Main aquatic activity**	Aquatic therapy Swimming training or instruction	36 (48.0) 10 (13.3)
	Aquatic and emotional therapy	4 (5.3)
	Various types of activities	25 (33.3)

#### 4.1.2 The questionnaire's face validity

‐ The degree to which the questionnaire fits its goals:

No statistically significant between‐group (parents vs. instructors) differences were observed in the prevalence of each answer option (*p* range: .15–.90). Most respondents reported that the questionnaire fits its purpose “to a very large extent” (parents—61.3%, instructors—60.0%; *p* < .05)

‐ The possibility to complete the questionnaire (parents) or explain (instructor):

No statistically significant between‐group differences were observed in the prevalence of each answer option (*p* range: .29–.85). Within the parents, the prevalence of those reporting that the questionnaire was “to a very large extent” easy to follow (52.0%) was statistically significantly higher than each of the other response options (*p* < .05). Among the instructors, the prevalence of those reporting answer options 1 (“to a very large extent,” 46.7%) and 2 (36.0%) was statistically significantly greater than the prevalence observed in answer options 3–5 (*p* < .05).

‐ The instructors' ability to derive goals from the questionnaire:

Most instructors (chi‐squared = 35.98, *p* < .001) reported that they were able to derive goals from the questionnaire to a very large extent (73.3%). Other chosen options: 2–18.7%, 3–6.7%, 4–1.3%, and 5–0%.

‐ The nature of the questionnaire (in terms of recommendations from participants for changes in the questionnaire):

The majority of the parents and instructors replied that they had nothing to add or change (86.7% of the sample). Among the other answers, the most prominent change recommendations focused on two main issues: (a) the questions are too long or general (13.3% of the sample), and (b) not enough references to disabilities that are not physical (behavioral, emotional, or communicative (10.6%).

Among the instructors, 88% of the instructors did not have any suggestions for change. The two most common suggestions were (a) adding additional health conditions (e.g., emotional domain, sensory regulation, behavior) to 12% of the sample and (b) that the questionnaire is too long (9.3%).

‐ Time to complete the questionnaire:

The most prevalent time frame to complete the questionnaire was 21–30 min (32% of the sample) followed by 31–40 min (22.7%). The other 45% of the sample required 0–20 min (13.3%), 41–50 min (13.3% of the sample), and 51–60 min (4.0% of the sample).

#### 4.1.3 The questionnaire's internal and interrater reliability

‐ Internal reliability—Part 2: ICF's A&P categories

Cronbach's alpha of higher or equal to .9 was found for all the questionnaires’ domains in both parents' and instructors' reports (Table 4).

**TABLE 4:** Questionnaire's internal reliability—Part 2: ICF's A&P categories.

Domain evaluation	Question number	Number of items	Cronbach's alpha	
			Instructor	Parents
**d4554—Swimming**	1	4	.97	.99
**d155—Acquiring skills**	2	4	.98	.98
**d160—Focusing attention**	3	3	.98	.99
**d175—Solving problems**	4	4	.99	.99
**d177—Making decisions**	5	3	.98	.98
**d210—Undertaking a single task**	6	3	.99	.97
**d250—Managing one's own behavior**	7	5	.99	.98
**d350—Conversation**	8	4	.93	.92
**d410—Changing basic body positions**	9	10	.92	.90
**d415—Maintaining a body position**	10	6	.90	.91
**d445—Hand and arm use**	11	5	.96	.96
**d450—Walking**	12	9	.96	.94
**d455—Moving around**	13	7	.93	.90
**D460—Moving around in different locations**	14	5	.99	.98
**d520—Caring for body parts**	15	6	.90	.90
**d540—Dressing**	16	9	.92	.90
**d710—(Basic) interpersonal interactions**	17	5	.94	.91
**d7504—Informal relationships with peers**	18	4	.93	.92
**d760—Family relationships**	19	5	.92	.91
**d880—Engagement in play**	20	6	.94	.90
				.91
**D920—Recreation and leisure**	21	9	.90	
**Goals' questionnaire**	1	21	.97	.98

 

‐ Interrater reliability (intraclass correlation coefficient)—Part 2: ICF's A&P categories

Out of the 116 categories within the 21 domains, 64 categories had excellent reliability (.9 < ICC), 39 a good reliability (.75 < ICC < .9), 8 with moderate reliability (.5 < ICC < .75), and only 5 categories had poor reliability (ICC < .5; Annex 2).

‐ Interrater reliability—Part 3: ability to set future goals for AA

Out of the 21 domains, 13 domains had an excellent reliability (.9 < ICC), 5 with a good reliability (.75 < ICC < .9), and only 3 with a moderate reliability (.5 < ICC < .75; Table 5).

**TABLE 5:** Questionnaire's interrater reliability—Part 3: the ability to set future goals for the AA.

Item	Intraclass correlation coefficient	Item	Intraclass correlation coefficient
1	.98	12	.95
2	.96	13	.53
3	.88	14	.90
4	.93	15	.61
5	.81	16	.86
6	.90	17	.91
7	.91	18	.93
8	.90	19	.76
9	.91	20	.89
10	.94	21	.97
11	.61		

*Note*: values between .5 and .75 indicate moderate reliability (denoted in orange), values between .75 and .9 indicate good reliability (denoted in yellow), and values greater than .90 indicate excellent reliability (denoted in green).

 

‐ Interrater reliability—differences in total scores
‐ Differences between instructor and parents in Part 2: ICF's A&P categories—total score—no statistically significant differences were observed between instructors and parents in total ICF score (Annex 3) (statistic *t* = −0.44, *p* = .65).
‐ Differences between instructor and parents in Part 3, ability to set future goals for AA—total score—despite the similar scores, statistically significant between‐group differences were observed in the total goal score (Annex 4). More specifically, the instructor's scores were significantly higher than those of the parents, suggesting that the instructors recommended significantly more goals than the parents (statistic *t* = −2.08, *p* = .04).

## 5 DISCUSSION

From the results, it can be concluded that the new questionnaire is valid and reliable for use to obtain comprehensive information about the children before AA, both through self‐filling by the parents or in response to the instructors' interview. The questionnaire allows the instructors to set goals for AA.

### 5.1 The nature of the questionnaire

‐ Among the two groups, there was agreement that the questionnaire fits its purpose “to a very large extent” (parents—61.3%, instructors—60.0%; *p* < .05).
‐ The questionnaire was found to be easy to fill out or explain to a large extent (52.0% of the parents; and 46.7% of the instructors, respectively).
‐ The time for the instructors to fill out the questionnaire ranged from 21 to 60 min; 54.6% of the instructors reported a time between 21 and 40 min.
‐ The majority of the participants had nothing to add or change in the questionnaire (86.7% of the sample). Among the suggestions for a change, the two most common suggestions were: adding additional domains concerning other health conditions and getting the questionnaire shorter.

The questionnaire's structure allows the instructors to get much information about the child, information that covers all areas of the child's life: the first part provides general information including the child's health condition, age, family and social situation, education, and compliance with academic requirements, previous experience in AE, personal characteristics and the child's quality of life.

The second part covers the child's functioning and social participation abilities. These areas are especially important for setting AA goals since AA is an activity with many effects on the child physically, emotionally, and socially (Hadar‐Frumer et al., 2023).

Following the parents' and instructors’ responses regarding the nature of the questionnaire, we made several changes and adjustments to the final questionnaire such as:

‐ Going through the open questions in the first part and adapting them to the various comments.
‐ Adding space for verbal comments.
‐ In the second part—allowing the option to skip questions irrelevant to the child and refer only to those that are. This way, the duration of filling out the questionnaire is greatly shortened.

### 5.2 Internal and interrater reliability

‐ A high internal reliability was found between all the categories of each one of the questions. This indicates that these items belong to the same world of content. Cronbach's alpha ≥ .9 was found between the answers of the two groups, which increased the certainty of reliability in the similarity between the two groups’ answers and of a high consistency among each group's answers.
‐ As for the interrater reliability, excellent and good reliability (ICC values) was found in 103 of the 116 categories (88.7%) when only 4% (5 categories) had poor internal reliability. In total ICF scores (paired *t*‐tests) no statistically significant differences were observed between the two groups. This result indicates that the parents answered the same questions in the same way in the two separate situations (self‐filling and answering the instructor's questions). On the other hand, as for setting future goals for AA, a difference was found between the goals as determined by the parents compared to those determined by the instructors.
 Conclusions:
‐ The parents can answer the questionnaire unaccompanied before meeting the instructor.
‐ Setting the goals requires extensive knowledge, more than the functioning of the child, therefore this step requires the final recommendations from the instructor after discussing with the parent.

An interesting result emerges from the ICC analysis of the interrater reliability of the values of the 21 domains’ categories (Annex 2)—all three questions with very low correlation values (<.5) were from the domains of mobility: D410—Changing basic body positions; D415—Maintaining a body position; D445—Hand and arm use. These results are somewhat puzzling because the children are participating in AA which is mainly a motor activity and it is to be expected that the parents will be aware of the children's motor control. At the same time, looking at the children's data in the other motor areas and especially in mobility and walking, we found out that out of 25 children reported, 15 (%60) of the children could independently move around in the house and outside it, even in environments with various obstacles. We hypothesize that since these children were 6–12 years old and many of them were independent in mobility at the time of filling out the questionnaire, the parents were less focused on their children's ability to control in areas with an easier developmental requirement (those that were expressed in these categories) so that they may not have been 100% aware of control of their children in these specific areas.

A question that arises from that data is why there is such a high percentage of mobile children within the group who were tested regarding the reliability of the questionnaire (the “reliability group”). Is this a similar percentage to the general group of children who participated in the study or did those parents of children who are independent in walking tend to participate more easily in the research?

Comparing the percentages of independence in walking for the general group of children (75) and among the children of the “reliability group” (Table 6), it can be seen that relatively, in the general group, there is a higher percentage of children who are independent in walking than in the “reliability group.” Hence the assumption made is incorrect.

**TABLE 6:** The percentages of independence in walking for the general group of the study compared to the “reliability group.”

Group	Number of participants	Walk independently 5 steps forward Number (percentages)	Walk independently 5 steps backward Number (percentages)	Walk independently over 10 meters Number (percentages)	Walk independently in the house and outside it, even in environments with various obstacles Number (percentages)
Reliability Group	25	16 (64%)	16 (64%)	13 (52%)	15 (%60)
All children	75	61 (81.3%)	52 (69.3%)	58 (77.3%)	51 (68%)

This conclusion raises another questions: why, among the participants in the study, there is such a high percentage of independent‐walking children—are these percentages similar to the general population of children with DD aged 6–12, or the questionnaire is not adapted to the entire population of children with DD and therefore parents of children, who do not walk independently, chose not to participate?

Among children with typical development, independent walking (at least 5 steps without any help; Størvold et al., 2013) appears between the ages of 8.2–17.6 months, and 95% of children walk independently between the ages of 9 and 16 months (Størvold et al., 2013; WHO Multicentre Growth Reference Study Group, 2006; Bishop et al., 2016).

Among children with DD, there is a delay in this area as well, and the intensity of the delay depends greatly on the cause of the delay and its severity. For example:

For children with CP, reaching independent walking depends, in addition to age, on the type of CP (spastic unilateral, spastic bilateral, dyskinetic, and ataxic cerebral palsy), the presence of epilepsy, the IQ level, and severe visual or hearing impairments (Beckung et al., 2008). Beckung et al. (2008) in their survey of 9012 participants born between 1976 and 1996 found that 54% of them had experienced independent walking around the age of 5 years.

Palisano et al. (2010) linked the level of control in gross motor skills, as defined by the Gross Motor Function Classification System (GMFCS), and the age of reaching independent walking. they found that children with GMFCS I were already walking independently at age 3, among children with GMFCS II the age range was wider, and most of them (76%) could walk indoors independently already at the age of 4. For children with GMFCS III, the age of reaching independent walking ranges from 4 to 9 years, and among children with GMFCS IV‐V, the striking majority didn't reach independent walking and got around in wheelchairs (Palisano et al., 2010).

Regarding children on the autistic spectrum (ASD), Kokubun et al. (1995) found that by the age of 18 months, most of the children (93%) can already walk independently; the researchers also found that among children with Down syndrome, the age of reaching walking is higher—after the age of 2 years (Kokubun et al., 1995).

Reindal et al. (2020) examined the relationship between the age of the start of walking (Age Of Walking—AOW) and the severity of the onset of ASD; they defined late AOW as beyond the age of 16 months. Among the 490 children, who participated in this study, the AOW range was between 8 months and 4 years with a mean of 14.5 months (mean difference of 1.9 months compared to typical children in Norway according to Størvold et al. (2013)).

From these studies, it can be understood that between the ages of 6 and 12 (the age of the children in our research), most children can walk independently. Therefore, it seems that the questionnaire is suitable for the population of children of these ages. We recommend a follow‐up study in which the questionnaire will be tested specifically with children who do not walk, thereby validating its suitability for this population.

The important practical result is that the questionnaire was found to be valid and reliable for use among the parents without the guidance of an instructor or prior training. This means that parents can answer the questionnaires before the evaluation meeting, thus allowing the instructors to focus on a more accurate and practical evaluation of the child. Self‐filling of the questionnaire allows the parents to understand the purpose of the evaluation process, to be prepared for it, and to provide full and orderly answers.

These results and conclusions correspond with previous studies on questionnaires developed for parental self‐report (Illum & Gradel, 2017; O'Shea et al., 2023; Zieff et al., 2023; McCormack et al., 2010). All these researchers emphasize the importance of parents, as the “child experts,” who should be partners in the assessment and intervention processes: “No one is better qualified than parents to assess disabilities in their own children” [40, p.1].

### 5.3 Strengths, weaknesses, and recommendations

Among the questionnaires received, stood out the fact that most of the children have a relatively high level of functioning. This fact may affect the validity of the questions. For future studies we, therefore, recommend investigating children with additional levels of functioning such as walking and transferring, and adding a few more domains concerning the child's emotional factors, sensory regulation, behavior, etc. However, to ensure practicality in routine clinical settings, it is important not to expand on the time required to complete the questionnaire.

## 6 CONCLUSION

The developed questionnaire (AQUA CHILD) demonstrates good validity and reliability and serves as a valuable tool for establishing treatment goals when administered by both instructors and parents. Nonetheless, enhancing its effectiveness in capturing the child's overall ability and therapeutic objectives could be served by some more incorporating supplementary questions addressing nonphysical issues such as emotional and behavioral factors.

## AUTHOR CONTRIBUTIONS

**Merav Hadar Frumer**: Conceptualization; investigation. **Huib Ten Napel**: Conceptualization; visualization. **Maria José Yuste‐Sánchez**: Writing—review and editing. **Isabel Rodríguez‐Costa**: Investigation; supervision.

## CONFLICT OF INTEREST STATEMENT

The authors report there are no competing interests to declare.

## FUNDING

This research received no external funding.

### PEER REVIEW

The peer review history for this article is available at https://publons.com/publon/10.1002/brb3.70033.

## Supporting information



Supplementary Materials

Supplementary Materials

## Data Availability

Data sharing not applicable to this article as no datasets were generated or analyzed during the current study.
