# Supplementary material for: Design and validation of AQUA CHILD—Pre‐aquatic questionnaire assessing child development
Source: Brain Behav. 2024 Sep 29;14(10):e70033. doi: 10.1002/brb3.70033 (PMC11440034; doi:10.1002/brb3.70033)
Supplement: Supplementary file 1 — Supplementary Materials [file BRB3-14-e70033-s002.docx]

Annex – the questionnaire

**Evaluation of the child’s activity and participation in everyday life before aquatic activity - A Questionnaire for parents and caregivers**

**Instructions and Information**

- This questionnaire is about the Child’s functioning in daily and based on the domains of activity and participation of the ICF-CY (the International Classification of Functioning, Disabilities and Health of the World Health Organization - adapted for children).
- The questionnaire is intended for the purpose of gathering information about the daily-life functioning of children aged 6-12 in order to be able to set goals before beginning aquatic activities (AA) or at stages where a re-evaluation is desired.
- This information will allow the instructor to determine the abilities and challenges facing the child and to set, in collaboration with the parents, precise goals for the aquatic activity. Goals that aim to promote the child's functions in various aspects of daily life.
- The questionnaire can be filled in two ways:
- By self-filling of the parents before the assessment session.
- Through the instructor's interview with the parents at the assessment meeting.

**The questionnaire consists of six parts:**

1. Part 1: General information about the child - questions regarding the child’s personal and family information, everyday life activities, activities within the AE, Personal factors and QoL.
2. Part 2: Information regards the child’s abilities in various domains of daily activities.
3. Part 3: Summarizing the information and determining main goals for AA.
4. Part 4: Questions for the parents or caregivers regarding the relevance of the questionnaire to the child's evaluation procedure before the activity in the water and as part of a repeated evaluation - is the questionnaire clear and are there any topics to be added?
5. Part 5: Questions for the instructors regarding the relevance of the questionnaire to the child's evaluation process before the activity in the water and as part of a repeated evaluation and the time- is the questionnaire clear and are there topics to be added?
6. Part 6: Several general questions about the instructor.

- The questionnaire is currently in the process of construction and research as part of my PhD at the University of Alcalá in Madrid.
- **Answering the questionnaire constitutes consent to participation in the research**

Thank you very much for your cooperation and help.

Merav Hadar Frumer

**Part 1- general information about the child**

**Please mark the appropriate answers**

Date of completion --/--/----

The child's name................................ The parent/caregiver’s name...............................

1. **What is your connection to the child?**

- Mother
- Father
- Other…………………………..

1. **The child's gender:**

- Female
- Male
- Other…………………………..

1. **The child’s age:**

- 6
- 7
- 8
- 9
- 10
- 11
- 12

1. **The child’s medical diagnosis or condition:** …………………………………………………………………………………………………………………………………………
2. **Family status - number of siblings and the child's position among them:**

…………………………………………………………………………………………………………………………………………

1. **Educational system in which your child studies - grade/ general education system / special education system/ An integrated educational system:**

…………………………………………………………………………………………………………………………………………

1. **Does your child have difficulties functioning at school (academic, social)?**

…………………………………………………………………………………………………………………………………………

1. **Why did you choose this aquatic activity** **for your child?**

…………………………………………………………………………………………………………………………………………

1. **What are your expectations from this activity?**

…………………………………………………………………………………………………………………………………………

1. **Has the child ever participated in aquatic activities before?**

- Yes
- No

1. **How long does the child participate in the current aquatic activity?**

- First or second time
- 1-3 months
- 6 months
- 7-12 months
- 13-18 months
- more than 18 months

1. **What is the nature of the activity?**

- One-on-one activity
- Group activity
- Both activities

1. **In case your child has been participating for more than 3 months:**

**a. Do you know what the main goals of the activities are?**

- Yes
- No

**b. If yes, please describe them:**

…………………………………………………………………………………………………………………………………………

1. **Additional activities:**

**a. Does your child receive any additional therapy or participate in other activities?**

- Yes
- No

**b If yes, please describe the activity/ treatment:**

…………………………………………………………………………………………………………………………………………

**The following questions pertain to your child’s personal characteristics:**

1. **How would you describe your child’s usual disposition?**

- Calm, relaxed and smiling
- Restless - tends to get stressed easily
- Tends to be angry
- Apathetic

Remarks…………………………………………………………………………………………………………………………………

1. **Your child’s usual character:**

- Smiling
- Serious
- Upset
- Apathetic

Remarks………………………………………………………………………………………………..………………………………

1. **In which environment does your child function best?**

- Quiet environment
- Noisy environment
- No noticeable difference

Remarks…………………………………………………………………………………………………………………………………

1. **Motivation - to what degree does your child show initiative and participation in daily life?**

- Initiates activities
- Doesn’t tend to initiate but cooperates in activities initiated by others
- Doesn’t tend to initiate and cooperates with initiation by others after continued persuasion
- Resists initiative by others

Remarks……………………………………………………………………………………………..…………………………………

1. **Your child level of enjoyment of the aquatic environments (shower, pool, sea)?**

- Enjoys it and eager to enter the water
- Agrees to enter the water
- Enters the water after being persuaded
- Resists strongly to entering the water
- Apathetic

Remarks……………………………………………………………………………………..…………………………………………

1. **quality of life - how would you rate your child’s quality of life*?**

- Good
- Fair
- Poor
- I’m not sure

Remarks…………………………………………………………………………………………………………………………………

* **Quality of life** is defined by the World Health Organization as "the individual's perception of his/her position in life - in relation to his/her goals, expectations, standards and concerns".

Quality of life is not only related to the state of health. It can be referred to in terms of functioning or disability or in terms of a match between ambitions and results.

In children, the quality of life reflects the view of the child or the family regarding the effect of treatment, education, etc.;

**Part 2 - The child’s abilities in daily life**

**Guidelines:**

- The questionnaire is made up of 21 questions that represent different areas of the child's abilities in everyday life.
- Each question consists of several categories that describe the child's skill in this area.
- For each category you can choose one of the options offered in the top row (the coding scale).
- Each of the categories should be answered and the appropriate choice for the child's ability should be marked.
- It is important to address in the answer the best possible performance for the child in the specific activity - with or without supports and accessories.
- If the highest capacity is performed with any support, please state what it is in the comment’s line.
- If clarification is needed - additional descriptions of all activities in each domain as well as those that are not included in the domain, appear at the end of the questionnaire.

**1: Swimming -** Propelling the whole body through water by means of limb and body movements without taking support from the ground underneath, or the pool walls (d4554).

**Does your child swim at least 5 meters in the following ways:**

| **Coding/Skill** | **0** | **1** | **2** | **3** | **4** | **5** | **6** |
| --- | --- | --- | --- | --- | --- | --- | --- |
|  | **independent** | **with hand support** | **with full person’s support** | **with support of the pool’s floor or wall** | **only with flotation devices** | **with other support not specified** | **doesn't swim** |
| 1. Backstroke |  |  |  |  |  |  |  |
| 2. Crawl stroke |  |  |  |  |  |  |  |
| 3. Breast stroke |  |  |  |  |  |  |  |
| 4. Other swimming |  |  |  |  |  |  |  |
| **Remarks** for question 1 ……………………………………………………………………………………………………………….. | | | | | | | |

| **Coding/Skill** | **0** | **1** | **1** | **1** | **2** | **3** |
| --- | --- | --- | --- | --- | --- | --- |
|  | **Indepen- dent** | **with a person’s support** | **with assistive device** | **with both supports** | **not capable** | **not relevant** |
| **2: Acquiring skills**  Developing basic and complex competencies in integrated sets of actions or tasks (d155).  **Is your child able to acquire or develop new skills in daily activities such as:** | | | | | | |
| 1. Use new utensils for food |  |  |  |  |  |  |
| 2. Use work tools/tools from home for purposes other than their original purpose |  |  |  |  |  |  |
| 3. Develop a new game with games or toys around |  |  |  |  |  |  |
| **Remarks** for question 2 (including assistive devices)……………………………………………………………………………….. | | | | | | |
|  | | | | | | |
| **3: Focusing attention -** Intentionally focusing on specific stimuli (d160)  **Can your child purposefully focus on a task by filtering out distracting noises, to achieve:** | | | | | | |
| 1. Focusing attention on the person in contact with him - on his instructions or requests |  |  |  |  |  |  |
| 2. Focusing on the task while ignoring the distracting stimuli in the environment |  |  |  |  |  |  |
| **Remarks** for question 3 (including assistive devices)……………………………………………………………………………….. | | | | | | |
|  | | | | | | |
| **4. Problem solving -** finding solutions to questions or situations in the child's daily life (d175)  **When a problem arises can your child:** | | | | | | |
| 1. Identify what the problem is and define it |  |  |  |  |  |  |
| 2. Propose possible solutions and ways to solve it |  |  |  |  |  |  |
| 3. Discuss with others and evaluate the possible impact of the solutions |  |  |  |  |  |  |
| 4. Execute the chosen solution |  |  |  |  |  |  |
| **Remarks** for question 4 (including assistive devices)……………………………………………………………………………….. | | | | | | |
|  | | | | | | |
| **5. Making decisions -** Making a choice among several options (177d).  **When there are several options can your child:** | | | | | | |
| 1. Choose a certain item/game to perform the required action |  |  |  |  |  |  |
| 2. Make a decision from among several similar options |  |  |  |  |  |  |
| 3. To perform one task from among several tasks that were raised as possible |  |  |  |  |  |  |
| **Remarks** for question 5 (including assistive devices)……………………………………………………………………………….. | | | | | | |
|  | | | | | | |
| **6. Performing a single task -** performing simple or complex actions, mentally or physically, individually or in a group (210d).  **When your child is asked to perform a single task (for example, "spread bread with butter" or " to throw a ball to a friend 10 times "), does he/she manage to complete the task while performing the following steps (please mark also whether he prefers to perform alone or in a group):** | | | | | | |
| 1. Initiate the task (alone/in a group) |  |  |  |  |  |  |
| 2. Organize what is needed - materials or a place to do the task (alone/in a group) |  |  |  |  |  |  |
| 3. Complete the task (alone/in a group) |  |  |  |  |  |  |
| **Remarks** for question 6 (including assistive devices)……………………………………………………………………………….. | | | | | | |
|  | | | | | | |
| **7. Managing one’s own behavior -** Carrying out coordinated (simple or complex) actions in a consistent manner in response to new situations, persons or experiences. (d250)  **What are your child's typical reactions in situations that are new to him/her - does he/she manage to maintain appropriate and consistent behavior and expression of emotions in situations such as:** | | | | | | |
| 1. Stay in a new environment |  |  |  |  |  |  |
| 2. Meet new classroom requirements |  |  |  |  |  |  |
| 3. Get close to new people |  |  |  |  |  |  |
| 4. Adjust the level of activity to the situation (for example, be quiet in class when required) |  |  |  |  |  |  |
| 5. Change from one state to another - like returning from a break to class |  |  |  |  |  |  |
| **Remarks** for question 7 (including assistive devices)……………………………………………………………………………….. | | | | | | |
|  | | | | | | |
| **8. Conversation -** starting, maintaining and ending an exchange of thoughts and ideas, with one or more familiar people, or a stranger, in a formal or casual situation (d350)  **Does your child hold a conversation with familiar people or strangers? If so - what means does he use?** | | | | | | |
| 1. Spoken language |  |  |  |  |  |  |
| 2. Written language |  |  |  |  |  |  |
| 3. Sign language |  |  |  |  |  |  |
| 4. Another form of conversation |  |  |  |  |  |  |
| **Remarks** for question 8 (including assistive devices)……………………………………………………………………………….. | | | | | | |
|  | | | | | | |
| **9. Changing basic body positions (d410 )**  **Can your child Get into and out of a certain body position and move from one location to another such as:** | | | | | | |
| 1. Lying down and rolling from side to side |  |  |  |  |  |  |
| 2. Transition from lying down to sitting |  |  |  |  |  |  |
| 3. Transition from sitting to lying down |  |  |  |  |  |  |
| 4. Transition from sitting on a chair/bed to standing |  |  |  |  |  |  |
| 5. Transition from standing to sitting on a chair/bed |  |  |  |  |  |  |
| 6. Transition from standing to sitting on the floor |  |  |  |  |  |  |
| 7. Transition from sitting on the floor to standing |  |  |  |  |  |  |
| 8. Entering and exiting positions of kneeling or sitting bent over |  |  |  |  |  |  |
| 9. Bending and moving the body's center of gravity |  |  |  |  |  |  |
| **Remarks** for question 9 (including assistive devices)……………………………………………………………………………….. | | | | | | |
|  | | | | | | |
| **10. Maintaining a body position (d415).**  **Does your child maintain body position for at least 5 seconds as required in a certain situation, such as:** | | | | | | |
| 1. Maintaining the head position |  |  |  |  |  |  |
| 2. Maintaining a supine position |  |  |  |  |  |  |
| 3. Maintaining a squatting position |  |  |  |  |  |  |
| 4. Maintaining a kneeling position |  |  |  |  |  |  |
| 5. Maintaining a sitting position |  |  |  |  |  |  |
| 6. Maintaining a standing position |  |  |  |  |  |  |
| **Remarks** for question 10 (including assistive devices)…………………………………………………………………………….. | | | | | | |
|  | | | | | | |
| **11. Hand and arm use (d445)**  **Does your child use hands and arms to perform coordinated actions required to move objects or to manipulate objects. For example:** | | | | | | |
| 1. Turning/pressing door handles to open them |  |  |  |  |  |  |
| 2. Throwing or catching an object |  |  |  |  |  |  |
| 3. Pulling or pushing objects |  |  |  |  |  |  |
| 4. Reaching out to a person or object |  |  |  |  |  |  |
| **Remarks** for question 11 (including assistive devices)…………………………………………………………………………….. | | | | | | |
|  | | | | | | |
| **12. Walking (450d).**  **Does your child progress by walking along a surface, step by step, so that one foot is always placed on the ground, in the following ways:** | | | | | | |
| 1. walk forward - 5 steps |  |  |  |  |  |  |
| 2. walking back - 5 steps |  |  |  |  |  |  |
| 3. walking sideways - 5 steps to the right |  |  |  |  |  |  |
| 4. walking sideways - 5 steps to the left side |  |  |  |  |  |  |
| 5. Walking a short distance - up to 10 meters |  |  |  |  |  |  |
| 6. Walking a distance of over 10 meters |  |  |  |  |  |  |
| 7. Walking on different surfaces - sand, grass, pavement |  |  |  |  |  |  |
| 8. Walking around obstacles - at home, outside |  |  |  |  |  |  |
| **Remarks** for question 12 (including assistive devices)…………………………………………………………………………….. | | | | | | |
|  | | | | | | |
| **Movement in space -** general mobility (not walking) (455d)  **Is your child mobile by means other than walking, such as:** | | | | | | |
| 1. Running / jogging at home or outside |  |  |  |  |  |  |
| 2. Jumping |  |  |  |  |  |  |
| 3. Crawling |  |  |  |  |  |  |
| 4. Climbing obstacles |  |  |  |  |  |  |
| 5. Mobility on a manual wheelchair |  |  |  |  |  |  |
| 6. Mobility on an electric wheelchair/scooter or other electric vehicle |  |  |  |  |  |  |
| **Remarks** for question 13 (including assistive devices)…………………………………………………………………………….. | | | | | | |
|  | | | | | | |
| **14. Moving around in different locations -** Walking and moving around in various places and situations (d460)  **Is your child mobile in various environments at home or outside (it doesn't matter which mode of mobility - the difference is the environment):** | | | | | | |
| 1. Moving between rooms in the house |  |  |  |  |  |  |
| 2. Moves inside other buildings, except the house |  |  |  |  |  |  |
| 3. Moves around the school - classroom and yard |  |  |  |  |  |  |
| 4. Moving on the street in the city |  |  |  |  |  |  |
| **Remarks** for question 14 (including assistive devices)…………………………………………………………………………….. | | | | | | |
|  | | | | | | |
| **15. Caring for body parts (d520)**  **Does your child manage to take care of the different parts of the body - skin, face, teeth, scalp, nails, etc., which require more than washing and drying, such as:** | | | | | | |
| 1. Care for skin – applying cream or bandage |  |  |  |  |  |  |
| 2. Brush teeth |  |  |  |  |  |  |
| 3. Comb and gather the hair |  |  |  |  |  |  |
| 4. Blow the nose |  |  |  |  |  |  |
| 5. Cut nails |  |  |  |  |  |  |
| **Remarks** for question 15 (including assistive devices)…………………………………………………………………………….. | | | | | | |
|  | | | | | | |
| **Dressing (d540)** - performing coordinated actions and tasks of dressing and removing clothes and shoes in sequence and in accordance with the weather and social conditions.  **Does your child wear and undress:** | | | | | | |
| 1. Shirts, blouses |  |  |  |  |  |  |
| 2. Pants, skirts |  |  |  |  |  |  |
| 3. Underwear, tights |  |  |  |  |  |  |
| 4. Hats |  |  |  |  |  |  |
| 5. Gloves |  |  |  |  |  |  |
| 6. coats |  |  |  |  |  |  |
| 7. Shoes, boots, sandals and slippers |  |  |  |  |  |  |
| 8. Splints/ prostheses |  |  |  |  |  |  |
| **Remarks** for question 16 (including assistive devices)…………………………………………………………………………….. | | | | | | |
|  | | | | | | |
| **17. (Basic) interpersonal interactions -** Interacting with people in a contextually and socially appropriate manner (d710)  **Does your child act appropriately in social situations such as:** | | | | | | |
| 1. Demonstrating consideration and appreciation for another |  |  |  |  |  |  |
| 2. Appropriate response to the feelings of others |  |  |  |  |  |  |
| 3. Demonstration of respect, warmth and tolerance |  |  |  |  |  |  |
| 4. Use of appropriate physical contact |  |  |  |  |  |  |
| **Remarks** for question 17 (including assistive devices)…………………………………………………………………………….. | | | | | | |
|  | | | | | | |
| **18. Informal relationships with peers (d7504).**  **Does your child manage to create and maintain informal relationships (conversation, play, etc.) with children in different social situations:** | | | | | | |
| 1. Children from the class |  |  |  |  |  |  |
| 2. Children from the sport and leisure activities |  |  |  |  |  |  |
| 3. Children from the neighborhood |  |  |  |  |  |  |
| **Remarks** for question 18 (including assistive devices)…………………………………………………………………………….. | | | | | | |
|  | | | | | | |
| **19. Family relations (d760).**  **Does your child manage to create and maintain close relationships (communication, play, etc.) with:** | | | | | | |
| 1. The parents |  |  |  |  |  |  |
| 2. Brothers |  |  |  |  |  |  |
| 3. Grandparents |  |  |  |  |  |  |
| 4. Uncles, aunts and cousins |  |  |  |  |  |  |
| **Remarks** for question 19 (including assistive devices)…………………………………………………………………………….. | | | | | | |
|  | | | | | | |
| **20. Engagement in play -** Purposeful, sustained engagement in activities with objects, toys, materials or games- individually or in a group. (d880)  **Which of the following describes your child's favorite play activity:** | | | | | | |
| 1. Game alone with the games around |  |  |  |  |  |  |
| 2. Watching the game of others with interest (observation) |  |  |  |  |  |  |
| 3. Playing at the same time as others without actual cooperation |  |  |  |  |  |  |
| 4. Playing with others in pair cooperation |  |  |  |  |  |  |
| 5. Playing with others in cooperation in a group over 2 children |  |  |  |  |  |  |
| **Remarks** for question 20 (including assistive devices)…………………………………………………………………………….. | | | | | | |
|  | | | | | | |
| **21. Recreation and leisure (d920)**  **What does your child like to do in his free time:** | | | | | | |
| 1. Participation in physical activity |  |  |  |  |  |  |
| 2. Engaging in art or creative hobbies |  |  |  |  |  |  |
| 3. Reading for pleasure |  |  |  |  |  |  |
| 4. Playing a musical instrument |  |  |  |  |  |  |
| 5 Participation in trips |  |  |  |  |  |  |
| 6. Participation in organized or private social activity |  |  |  |  |  |  |
| 7. Watching TV |  |  |  |  |  |  |
| 8. Playing with animals |  |  |  |  |  |  |
| **Remarks** for question 21 (including assistive devices)…………………………………………………………………………….. | | | | | | |

**Part 3: setting goals**

**Can you write down the goals that you want to promote for the child in the coming months?**

**Step 1:** Please circle the important areas for promotion in AA for the next three months (up to 4 areas including swimming).

| Domain’s number | Domain’s name |  | Domain’s number | Domain’s name |
| --- | --- | --- | --- | --- |
| 1 | Swimming |  | 12 | Walking |
| 2 | Acquiring skills |  | 13 | Moving around |
| 3 | Focusing attention |  | 14 | Moving around in different locations |
| 4 | Solving problems |  | 15 | Caring for body parts |
| 5 | Making decisions |  | 16 | Dressing |
| 6 | Undertaking a single task |  | 17 | (Basic) interpersonal interactions |
| 7 | Managing one’s own behavior |  | 18 | Informal relationships with peers |
| 8 | Conversation |  | 19 | Family relationships |
| 9 | Changing basic body positions |  | 20 | Engagement in play |
| 10 | Maintaining a body position |  | 21 | Recreation and leisure |
| 11 | Hand and arm use |  |  |  |

**Step 2:** Selected goals for the next 3 months (taking into account parents' expectations):

| Goal Number | Selected area (out of the 21 domains) | Specific skill for promotion (the skill’s verbal description) | Current ability of the child | A goal to achieve in 3 months |
| --- | --- | --- | --- | --- |
| 1 |  |  |  |  |
| 2 |  |  |  |  |
| 3 |  |  |  |  |
| 4 | swimming |  |  |  |

Remarks:………………………………………………………………………………………………………………………………

**Part 4: General comments – for the parent/caregiver**

**1.a. Was the questionnaire easy to follow and understand?**

Please mark: 1 -to a very large extent- 2 – 3 – 4 - 5-Not at all

| - 1 | - 2 | - 3 | - 4 | - 5 |
| --- | --- | --- | --- | --- |

**1.b. If Not – What would you change?**

………………………………………………………………………………………………………………………………………………

**2. Do you think the questionnaire fits its purpose - to provide comprehensive and appropriate information about your child's abilities in everyday life and in social life?**

Please mark: 1 -to a very large extent- 2 – 3 – 4 - 5-Not at all

| - 1 | - 2 | - 3 | - 4 | - 5 |
| --- | --- | --- | --- | --- |

**3. Do you want to add additional functions that are important for recognizing your child's abilities? If so - please list them here.**

………………………………………………………………………………………………………………………………………………

**4. Would you like to add anything else?**

………………………………………………………………………………………………………………………………………………

Date of completion --/--/----

The child's name...................................

Parent’s/Caregiver’s name and signature ……………………………...

**Part 5: General comments – for the instructors**

**1.a. Was the questionnaire easy to explain?**

Please mark: 1 -to a very large extent- 2 – 3 – 4 - 5-Not at all

| - 1 | - 2 | - 3 | - 4 | - 5 |
| --- | --- | --- | --- | --- |

**1.b. If Not – What would you change?**

…………………………………………………………………………………………………………………………………………………………….

**2. Do you think the questionnaire fits its purpose - to provide comprehensive and appropriate information about your child's abilities in everyday life and in social life?**

Please mark: 1 -to a very large extent- 2 – 3 – 4 - 5-Not at all

| - 1 | - 2 | - 3 | - 4 | - 5 |
| --- | --- | --- | --- | --- |

**3. Do you want to add additional functions that are important for in order to determine the child’s activity goals? If so - please list them here.**

………………………………………………………………………………………………………………………………………………

**4.a. Is it possible to derive goals for aquatic activity from the questionnaire?**

Please mark: 1 -to a very large extent- 2 – 3 – 4 - 5-Not at all

| - 1 | - 2 | - 3 | - 4 | - 5 |
| --- | --- | --- | --- | --- |

**4.b. If Not – What would you change?**

………………………………………………………………………………………………………………………………………………

**5. Would you like to add anything else?**

………………………………………………………………………………………………………………………………………………

6. **How long did it take you to complete the questionnaire (minutes)?**

………………………………………

**Part 6: General details about the instructor**

**1. What is your main Profession:**

………………………………………………………………………………………………………………………………………………

**2. How many years have you been working in the aquatic atmosphere?**

………………………………………

**3. Is the activity individual or group?**

- Individual activity
- Group activity
- Both activities

**4. Your main aquatic activity with children focuses on:**

- Aquatic therapy
- Swimming training or swimming instruction
- Emotional therapy
- Some of these areas
- Another area……………………………………..

Date of completion --/--/----

The child's name...................................

Instructor's/Parent’s/Caregiver’s name and signature ……………………………...

**Full definition of the domains according to the ICF-CY**

World Health Organization 2007

|  | **ICF Code** | **ICF Domain** | **Domain’s wordings (category)** | **Inclusions** | **Exclusion** |
| --- | --- | --- | --- | --- | --- |
| **1** | **d4554** | Swimming | Propelling the whole body through water by means of limb and body movements without taking support from the ground underneath. |  |  |
| **2** | **d155** | Acquiring skills | Developing basic and complex competencies in integrated sets of actions or tasks so as to initiate and follow through with the acquisition of a skill, such as manipulating tools or toys, or playing games. | acquiring basic and complex skills | learning to write (d145) and writing (d170), learning to play (d131) |
| **3** | **d160** | Focusing attention | Intentionally focusing on specific stimuli, such as by filtering out distracting noises. | Focusing attention on the human touch, face and voice  Focusing attention to changes in the environment |  |
| **4** | **d175** | Solving problems | Finding solutions to questions or situations by identifying and analyzing issues, developing options and solutions, evaluating potential effects of solutions, and executing a chosen solution such as in resolving a dispute between two people. | solving simple (single issue or question) and complex problems (multiple and interrelated issues) | thinking (d163); making decisions (d177) |
| **5** | **d177** | Making decisions | Making a choice among options, implementing the choice, and evaluating the effects of the choice, such as selecting and purchasing a specific item, or deciding to undertake and undertaking one task from among several tasks that need to be done. |  | thinking (d163); solving problems (d175) |
| **6** | **d210** | Undertaking (a single) task | Carrying out simple or complex and coordinated actions related to the mental and physical components of a single task, such as initiating a task, organizing time, space and materials for a task, pacing task performance, and carrying out, completing and sustaining a task. | undertaking a simple or complex task; undertaking a single task independently or in a group | acquiring skills (d155); solving problems (d175); making decisions (d177); undertaking multiple tasks (d220) |
| **7** | **d250** | Managing one’s own behavior | Carrying out simple or complex and coordinated actions in a consistent manner in response to new situations, persons or experiences, such as being quiet in a library. | Accepting novelty -  Responding to demands  Approaching persons or situations  Acting predictably  Adapting activity level |  |
| **8** | **d350** | Conversation | Starting, sustaining and ending an interchange of thoughts and ideas, carried out by means of spoken, written, sign or other forms of language, with one or more persons one knows or who are strangers, in formal or casual settings. | starting, sustaining and ending a conversation; conversing with one or many people |  |
| **9** | **d410** | Changing basic body position | Getting into and out of a body position and moving from one location to another, such as rolling from one side to the other, sitting, standing, getting up out of a chair to lie down on a bed, and getting into and out of positions of kneeling or squatting. | changing body position from lying down, from squatting or kneeling, from sitting or standing, bending and shifting the body’s center of gravity | transferring oneself (d420) |
| **10** | **d415** | Maintaining a body position | Staying in the same body position as required, such as remaining seated or remaining standing for work or school. | maintaining a lying, squatting, kneeling, sitting and standing position and head position |  |
| **11** | **d445** | Hand and arm use | Performing the coordinated actions required to move objects or to manipulate them by using hands and arms, such as when turning door handles or throwing or catching an object. | pulling or pushing objects; reaching; turning or twisting the hands or arms; throwing; catching | fine hand use (d440) |
| **12** | **d450** | Walking | Moving along a surface on foot, step by step, so that one foot is always on the ground, such as when strolling, sauntering, walking forwards, backwards, or sideways. | walking short (less than a KM) or long distances (more than a KM); walking on different surfaces; walking around obstacles | transferring oneself (d420); moving around (d455) |
| **13** | **d455** | Moving around | Moving the whole body from one place to another by means other than walking, such as climbing over a rock or running down a street, skipping, scampering, jumping, somersaulting or running around obstacles. | crawling, climbing, running, jogging, jumping, swimming, scooting, rolling and shuffling | transferring oneself (d420); walking (d450) |
| **14** | **d460** | Moving around in different locations | Walking and moving around in various places and situations, such as walking between rooms in a house, within a building, or down the street of a town. | moving around within the home, crawling or climbing within the home; walking or moving within buildings other than the home, and outside the home and other buildings |  |
| **15** | **d520** | Caring for body parts | Looking after those parts of the body, such as skin, face, teeth, scalp, nails and genitals, that require more than washing and drying. | caring for skin, teeth, hair, finger and toe nails, and nose | washing oneself (d510); toileting (d530) |
| **16** | **d540** | Dressing | Carrying out the coordinated actions and tasks of putting on and taking off clothes and footwear in sequence and in keeping with climatic and social conditions, such as by putting on, adjusting and removing shirts, skirts, blouses, pants, undergarments, saris, kimono, tights, hats, gloves, coats, shoes, boots, sandals and slippers. | putting on or taking off clothes and footwear and choosing appropriate clothing |  |
| **17** | **d710** | Basic interpersonal interactions | Interacting with people in a contextually and socially appropriate manner, such as by showing consideration and esteem when appropriate, or responding to the feelings of others. | showing respect, warmth, appreciation, and tolerance in relationships; responding to criticism and social cues in relationships; and using appropriate physical contact in relationships |  |
| **18** | **d7504** | informal relationships with peers | Creating and maintaining informal relationships with people who share the same age, interest or other common feature. |  |  |
| **19** | **d760** | Family relationships | Creating and maintaining kinship relationships, such as with members of the nuclear family, extended family, foster and adopted family and step-relationships, more distant relationships such as second cousins, or legal guardians. | Parent-child relationships – The parents’ abilities  Child-parent relationships – The child’s abilities  Sibling relationships  Extended family relationships - cousins, aunts and uncles and grandparents. |  |
| **20** | **d880** | Engagement in play | Purposeful, sustained engagement in activities with objects, toys, materials or games. occupying oneself or with others. | Solitary play  Onlooker play (observation)  Parallel play  Shared cooperative play |  |
| **21** | **d920** | Recreation and leisure | Engaging in any form of play, recreational or leisure activity, such as informal or organized play and sports, programmes of physical fitness, relaxation, amusement or diversion, going to art galleries, museums, cinemas or theatres; engaging in crafts or hobbies, reading for enjoyment, playing musical instruments; sightseeing, tourism and travelling for pleasure. | games, sports, arts and culture, crafts, hobbies and socializing | riding animals for transportation (d480); remunerative and nonremunerative work (d850 and d855); engagement in play (d880); religion and spirituality (d930); political life and citizenship (d950) |
