# Supplementary material for: Design and validation of AQUA CHILD—Pre‐aquatic questionnaire assessing child development
Source: Brain Behav. 2024 Sep 29;14(10):e70033. doi: 10.1002/brb3.70033 (PMC11440034; doi:10.1002/brb3.70033)
Supplement: Supplementary file 2 — Supplementary Materials [file BRB3-14-e70033-s001.docx]

***Annex 2*** *- Questionnaire's inter-rater reliability (intra-class correlation coefficient) – Part 2 – ICF’s A&P categories*

| **Intra-class correlation coefficient** | **Item** | | **Question**  **number** | **Intra-class correlation coefficient** | **Item** | **Question**  **number** | **Intra-class correlation coefficient** | **Item** | **Question**  **number** |
| --- | --- | --- | --- | --- | --- | --- | --- | --- | --- |
| 0.88 | A | | 18 | 0.88 | A | 11 | 0.91 | A | 1 |
| 0.91 | B | |  | 0.70 | B |  | 0.99 | B |  |
| 0.90 | C | |  | 0.67 | C |  | 0.98 | C |  |
| 1.00 | D | |  | 0.12 | D |  | 0.98 | D |  |
|  |  |  |  | 0.99 | E |  | 0.94 | A | 2 |
| 0.74 | A | | 19 | 0.82 | A | 12 | 0.96 | B |  |
| 0.98 | B | |  | 0.91 | B |  | 0.95 | C |  |
| 0.66 | C | |  | 0.83 | C |  | 0.98 | D |  |
| 0.73 | D | |  | 0.83 | D |  | 0.90 | A | 3 |
| 0.99 | E | |  | 0.87 | E |  | 0.95 | B |  |
|  |  |  |  | 0.88 | F |  | 0.99 | C |  |
| 0.87 | A | | 20 | 0.91 | G |  | 0.84 | A | 4 |
| 0.74 | B | |  | 0.92 | H |  | 0.97 | B |  |
| 0.87 | C | |  | 0.99 | I |  | 0.98 | C |  |
| 0.89 | D | |  | 0.93 | A | 13 | 0.89 | D |  |
| 0.93 | E | |  | 0.95 | B |  | 0.92 | A | 5 |
| 0.99 | F | |  | 0.88 | C |  | 0.95 | B |  |
| 0.89 | A | | 21 | 0.92 | D |  | 0.77 | C |  |
| 0.94 | B | |  | 0.99 | E |  | 0.96 | A | 6 |
| 0.98 | C | |  | 0.98 | F |  | 0.90 | B |  |
| 0.98 | D |  | | 0.99 | G |  | 0.92 | C |  |
| 0.96 | E |  | | 0.75 | A | 14 | 0.95 | A | 7 |
| 0.92 | F |  | | 0.79 | B |  | 0.87 | B |  |
| 0.96 | G |  | | 0.86 | C |  | 0.95 | C |  |
| 0.91 | H |  | | 0.86 | D |  | 0.96 | D |  |
| 0.98 | I |  | | 0.99 | E |  | 0.89 | E |  |
|  |  |  | | 0.89 | A | 15 | 0.78 | A | 8 |
|  |  |  | | 0.90 | B |  | 0.97 | B |  |
|  |  |  | | 0.97 | C |  | 0.97 | C |  |
|  |  |  | | 0.97 | D |  | 0.99 | D |  |
|  |  |  | | 0.97 | E |  | 0.50 | A | 9 |
|  |  |  | | 0.92 | F |  | 0.37 | B |  |
|  |  |  | | 0.86 | A | 16 | 0.36 | C |  |
|  |  |  | | 0.94 | B |  | 0.78 | D |  |
|  |  |  | | 0.92 | C |  | 0.78 | E |  |
|  |  |  | | 0.84 | D |  | 0.79 | F |  |
|  |  |  | | 0.95 | E |  | 0.33 | G |  |
|  |  |  | | 0.97 | F |  | 0.91 | H |  |
|  |  |  | | 0.90 | G |  | 0.92 | I |  |
|  |  |  | | 0.92 | H |  | 0.99 | J |  |
|  |  |  | | 0.84 | I |  | 0.80 | A | 10 |
|  |  |  | | 0.89 | A | 17 | 0.70 | B |  |
|  |  |  | | 0.81 | B |  | 0.89 | C |  |
|  |  |  | | 0.85 | C |  | 0.83 | D |  |
|  |  |  | | 0.91 | D |  | 0.46 | E |  |
|  |  |  | | 0.98 | E |  | 0.80 | F |  |

Notes: Values less than 0.5 are indicative of poor reliability (denoted in red), values between 0.5 and 0.75 indicate moderate reliability (denoted in orange), values between 0.75 and 0.9 indicate good reliability (denoted in yellow), and values greater than 0.90 indicate excellent reliability (denoted in green).

***Annex 3*** *– Figure 1.* *Differences between instructor and parents in ICF total score*


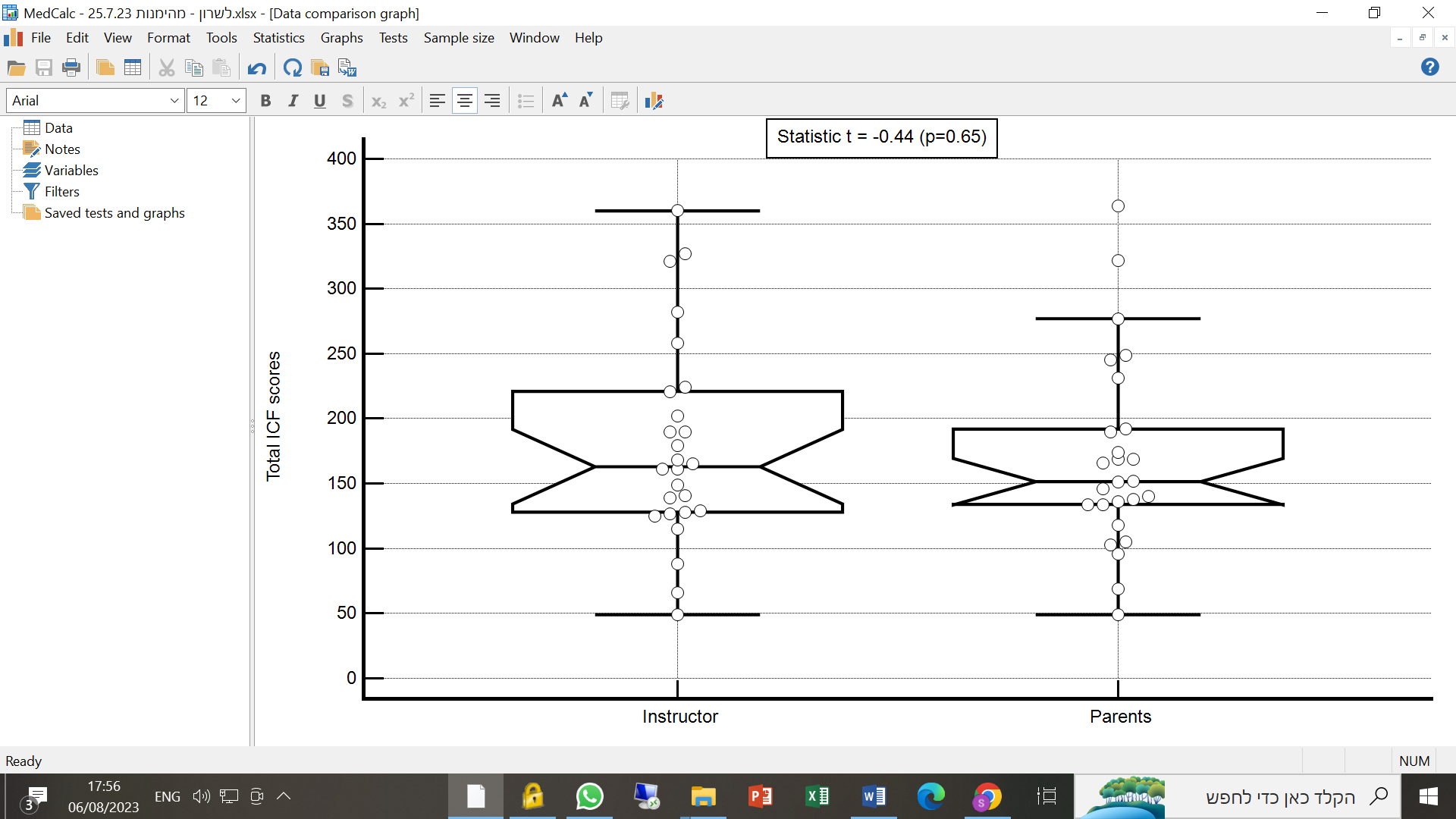


*Notes:* The central box represents the values from the lower to upper quartile (25-75 percentiles); the vertical line extends from the minimum to the maximum value, excluding outside values which are displayed as separate points. An outside value is defined as a value that is smaller than the lower quartile minus 1.5 times the interquartile range, or larger than the upper quartile plus 1.5 times the interquartile range; the middle line represents the median.

***Annex 4*** *– Figure 2. Differences between instructor and parents in goals total score*


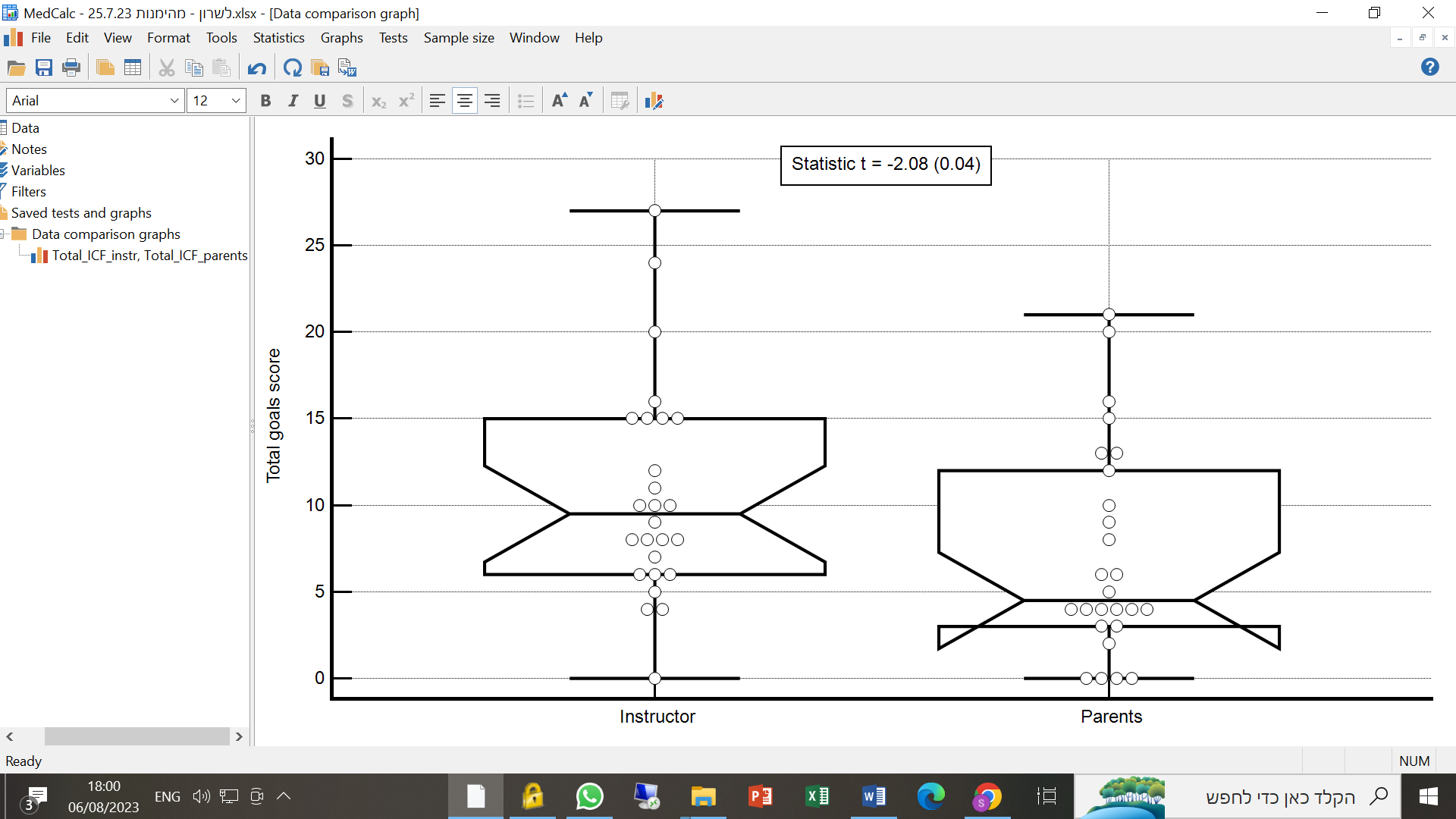


*Notes: The central box represents the values from the lower to upper quartile (25-75 percentiles); the vertical line extends from the minimum to the maximum value, excluding outside values which are displayed as separate points. An outside value is defined as a value that is smaller than the lower quartile minus 1.5 times the interquartile range, or larger than the upper quartile plus 1.5 times the interquartile range; the middle line represents the median.*
